# The Association of Macronutrient Consumption and BMI to Exhaled Carbon Dioxide in Lumen Users: Retrospective Real-World Study

**DOI:** 10.2196/56083

**Published:** 2024-04-01

**Authors:** Shlomo Yeshurun, Tomer Cramer, Daniel Souroujon, Merav Mor

**Affiliations:** 1 Metaflow Ltd Tel-Aviv Israel; 2 School of Public Health Tel Aviv University Tel-Aviv Israel

**Keywords:** app, applications, association, BMI, body mass index, carbohydrate, carbon dioxide, consumption, correlate, correlation, diet, dietary, exhalation, exhale, food, Lumen, macronutrient, meal, metabolic flexibility, metabolic, metabolism, mHealth, mobile health, nutrient, nutrition, nutritional, obese, obesity, postprandial, prandial, retrospective, weight

## Abstract

**Background:**

Metabolic flexibility is the ability of the body to rapidly switch between fuel sources based on their accessibility and metabolic requirements. High metabolic flexibility is associated with improved health outcomes and a reduced risk of several metabolic disorders. Metabolic flexibility can be improved through lifestyle changes, such as increasing physical activity and eating a balanced macronutrient diet. Lumen is a small handheld device that measures metabolic fuel usage through exhaled carbon dioxide (CO_2_), which allows individuals to monitor their metabolic flexibility and make lifestyle changes to enhance it.

**Objective:**

This retrospective study aims to examine the postprandial CO_2_ response to meals logged by Lumen users and its relationship with macronutrient intake and BMI.

**Methods:**

We analyzed deidentified data from 2607 Lumen users who logged their meals and measured their exhaled CO_2_ before and after those meals between May 1, 2023, and October 18, 2023. A linear mixed model was fitted to test the association between macronutrient consumption, BMI, age, and gender to the postprandial CO_2_ response, followed by a 2-way ANOVA.

**Results:**

The model demonstrated significant associations (*P*<.001) between CO_2_ response after meals and both BMI and carbohydrate intake (BMI: β=–0.112, 95% CI –0.156 to –0.069; carbohydrates: β=0.046, 95% CI 0.034-0.058). In addition, a 2-way ANOVA revealed that higher carbohydrate intake resulted in a higher CO_2_ response compared to low carbohydrate intake (*F*_2,2569_=24.23; *P*<.001), and users with high BMI showed modest responses to meals compared with low BMI (*F*_2,2569_=5.88; *P*=.003).

**Conclusions:**

In this study, we show that Lumen’s CO_2_ response is influenced both by macronutrient consumption and BMI. The results of this study highlight a distinct pattern of reduced metabolic flexibility in users with obesity, indicating the value of Lumen for assessing postprandial metabolic flexibility.

## Introduction

### Background

The presence of obesity is the leading risk factor for metabolic disorders such as type 2 diabetes (T2D) and cardiovascular diseases (CVDs), and it has been linked to a reduced life expectancy [1,2]. Metabolic syndrome represents a collection of metabolic abnormalities that includes obesity as well as insulin resistance, hypertension, and dyslipidemia [3]. Changing the macronutrient distribution, such as low-fat or low-carbohydrate diets, has been proposed for the treatment and management of metabolic syndrome [4], and they have been shown to improve several clinical features of metabolic syndrome, including weight loss and cardiovascular risk [5,6].

Metabolic flexibility is the ability to switch between fuel sources (such as carbohydrates and fats) in response to their availability and metabolic demands, which is crucial for maintaining overall health and well-being [7,8]. Lifestyle modifications, such as exercise and a balanced diet, can therefore enhance metabolic flexibility, and high metabolic flexibility is associated with improved health outcomes, including a reduced risk of metabolic syndrome [7]. In addition, it was found to be impaired in individuals with obesity and T2D compared to lean and healthy individuals [9,10]. Assessment of metabolic flexibility can be conducted through postprandial changes (following a meal or any other insulin stimulation) in the respiratory exchange ratio (RER) from the metabolic cart [11,12], which estimates the body’s preference for macronutrient oxidation. Accordingly, several studies showed different RER responses between participants with high metabolic flexibility compared with participants with low flexibility for fasting, a high-carbohydrate diet, and a high-fat diet [13]. A recent study has shown that RER is greater after high-carbohydrate overfeeding than high-fat overfeeding, and impaired metabolic flexibility, as measured by ΔRER, is associated with greater weight gain over the following 6- and 12-month periods [14]. Furthermore, ΔRER is significantly elevated after high-carbohydrate intake compared with high-fat intake, indicating that metabolic flexibility is influenced by macronutrient composition [15]. These studies show that metabolic flexibility can be assessed based on RER from the metabolic cart.

However, metabolic carts are expensive, time-consuming, and only available in health care laboratories [16]. Thus, a small handheld device was developed to measure metabolic fuel usage by analyzing exhaled carbon dioxide (CO_2_). The Lumen device is a portable breath-analyzer that measures metabolic fuel use through exhaled CO_2_ and was found to be in agreement with the RER measured by the metabolic cart [17]. Furthermore, Lumen was found to be able to detect different metabolic responses to low- or high-carb lifestyles through CO_2_ changes [18].

A variety of mobile health apps, including Lumen, which are designed for mobility and ubiquity, have demonstrated promising results in enhancing metabolism and facilitating weight management [19]. Using real-time data tracking, these tools empower individuals to make more informed health decisions [20]. As such, mobile apps such as Lumen have the potential to make a positive difference in people’s health and well-being [21].

### Objective

This study examined data collected from Lumen users’ exhaled CO_2_ measurements taken before and after logging a meal. The objective of this analysis was to investigate the postprandial response of Lumen’s CO_2_ measurements to meals logged by Lumen users and to understand the association of this response with macronutrient consumption as well as users’ age, gender, and BMI.

## Methods

### Ethical Considerations

This study was determined to be exempt from institutional board review (IRB) under category 2, as detailed in 45 CFR 46.104(d) and the standard operating procedure of the Biomedical Research Alliance of New York (BRANY), by the BRANY Social, Behavioral, and Educational Research (SBER) IRB on May 9, 2023 (BRANY IRB File 23-119-1476).

### Study Design

This is a retrospective observational study based on deidentified data collected from users of the Lumen device and app.

### Participants

Participants’ data were collected retrospectively between May 1, 2023, and October 18, 2023. All users in the analysis were aged 18 years or older. Since only 12 users who were underweight (BMI≤18.5) were found in the database, they were removed from the analysis.

### Data Sources

**Figure 1:**
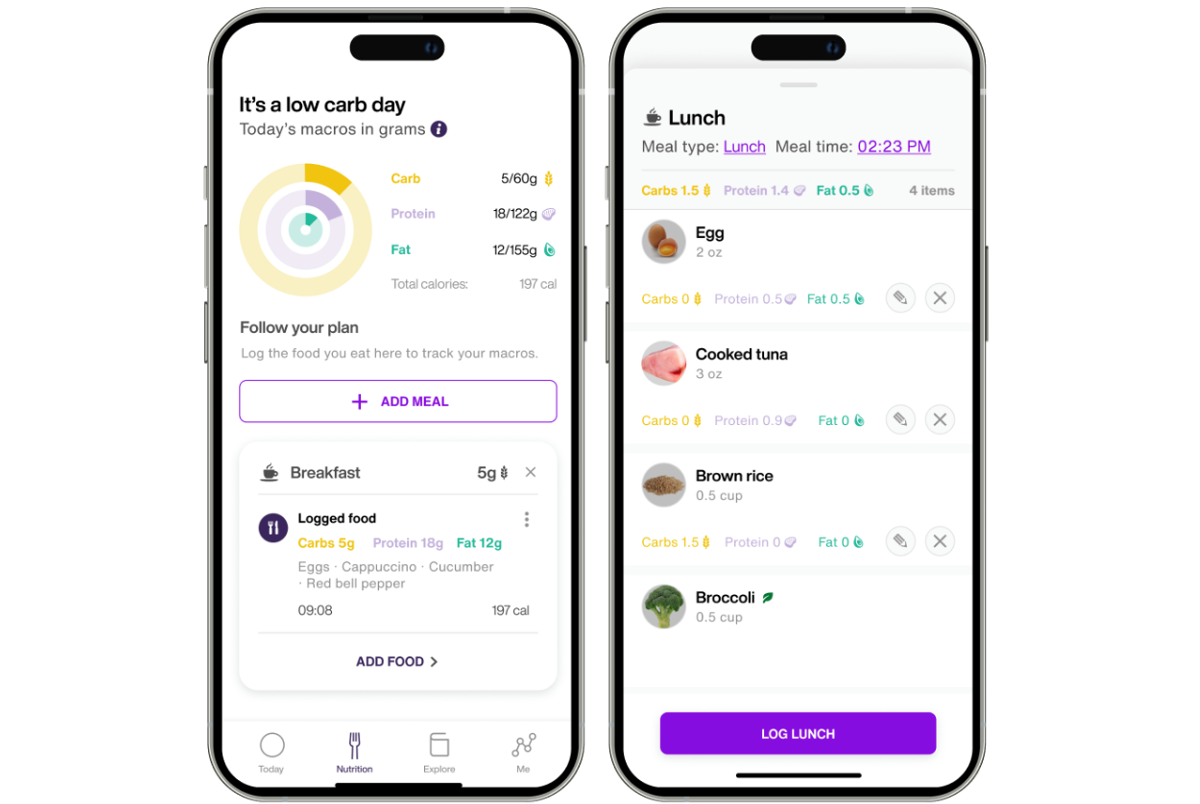
Lumen mobile app screenshots detailing the logged meals and their macronutrient distribution.

As part of the onboarding process, users were required to specify their gender, date of birth, height, and weight. The use of the Lumen app includes an optional morning fasted measurement with the Lumen device, as well as recommended pre- and postmeal measurements throughout the day and a bedtime measurement before sleeping. The app provides nutritional recommendations based on the user’s personal preferences as well as their morning CO_2_ measurement regarding the amount of macronutrients one should consume during the day. The app also allows users to log their meals—whether they are breakfast, lunch, dinner, or snacks. The macronutrient composition of these meals is determined by the nutritional database of Nutritionix [22], which includes all nutritional data available from the United States Department of Agriculture’s (USDA) Food Composition Database, restaurant chain data, and foods added by Nutritionix’s dietitians. An example of the food log feature in the app is shown in Figure 1.

Exhaled CO_2_ measurements were obtained using the Lumen device (Metaflow Ltd). The Lumen mobile app guides the user through each phase of the Lumen maneuver (inhale, breath hold, and exhale), as previously described [17].

In this study, premeal and fasting measurements were defined as “premeal %CO_2_,” while postmeal and bedtime measurements were considered to be the “postmeal %CO_2_” measure, with a maximum of 210 minutes between pre- and postmeal measurements selected for the analysis. The relative change in %CO_2_ from premeal to postmeal was calculated for the final analysis. In addition, meals that were tagged as either breakfast, lunch, or dinner were selected for the analysis, while snacks were removed. Further data were excluded from the analysis using the Tukey method for outlier removal with an IQR of 1.5 for all macronutrients.

### Statistical Analysis

Data were analyzed using Python JupyterLab (version 3.6.3; Project Jupyter), and all statistical analyses were conducted with the Python programming language, using custom scripts and the *statsmodels* package [23]. Figures were made with GraphPad Prism (version 10.1.0 for Windows, GraphPad Software) [24].

A linear mixed model (LMM) was fitted in order to test the relationship between the quantity of macronutrients (carbohydrates, fats, and proteins) and personal information (gender, age, and BMI) to the outcome variable of percentage of change in %CO_2_ from premeal to postmeal, where users’ unique ID was adjusted for as a random effect [25]. A 2-way ANOVA was used to test the differences between different groups of statistically significant variables in the LMM. Among all the analyses performed, a 2-sided *P*≤.05 was considered statistically significant.

## Results

### Participants

Overall, a total of 2607 users who completed 6671 coupled pre- and postmeal sessions from 6207 logged meals were used in the final analysis, with most users contributing only 1 session into the analysis (median 1, IQR 1-2 sessions per user).

### Descriptive

A total of 81.97% (2137/2607) of the participants were women, and men and women did not differ in their ages or BMIs. A summary of the characteristics of the participants in the study is presented in Table 1.

Additionally, the macronutrient composition of the meals consumed by the participants was primarily carbohydrates, followed by proteins and fats, as detailed in Table 2.

**Table 1:** Sample characteristics of users (N=2607).

Characteristics	Values
Gender (female), n (%)	2137 (81.97)
Age (years), mean (SD)	47.6 (9.4)
BMI (kg/m^2^), mean (SD)	28.8 (6.0)

**Table 2:** Macronutrient composition of meals (N=6207).

Parameters	Values, mean (SD)
Carbohydrates (grams)	32.5 (20.5)
Proteins (grams)	29.7 (14.7)
Fats (grams)	22.5 (13.7)

### Evaluation Outcomes

**Figure 2:**
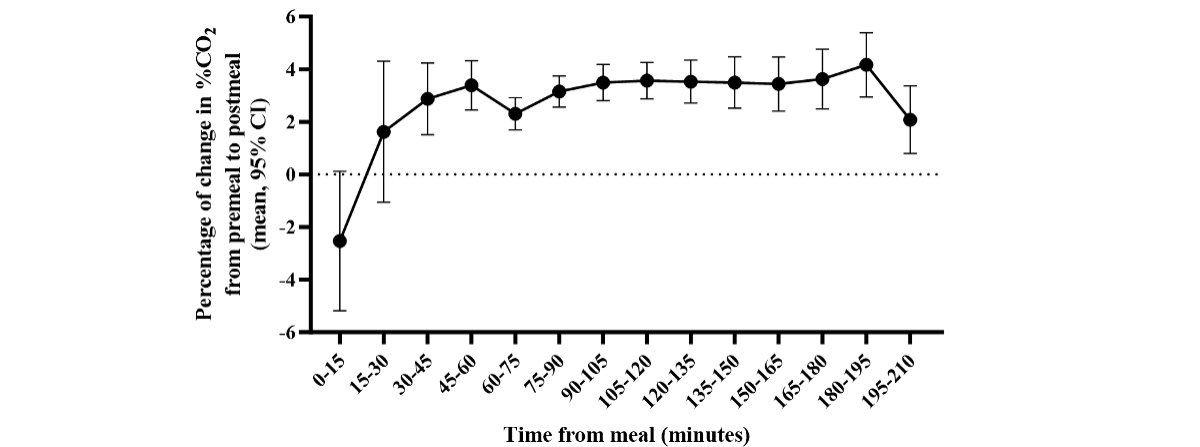
Exhaled carbon dioxide (CO_2_) response to meal at different time intervals (N=6671). Results represent mean (95% CI).

The overall CO_2_ response after meal consumption at various time points is shown in Figure 2. As expected, %CO_2_ measured by Lumen 30 to 210 minutes following meal intake increased significantly compared to premeal measurement.

The relationship between postprandial CO_2_ response and macronutrient intake, as recorded in the Lumen app and users' personal information, was examined using Linear Mixed Modeling (LMM). The users' unique ID was incorporated as a random effect in the analysis. The model revealed a statistically significant relationship between the CO_2_ response and the BMI of the users (*P*<.001), as well as their carbohydrate intake (*P*<.001). Lower BMI predicted a more significant increase in postprandial CO_2_ response, and increased carbohydrate intake predicted a greater postprandial CO_2_ response (Table 3). In contrast, their postprandial CO_2_ response was not significantly influenced by their age, gender, fat intake, or protein intake (all *P*>.05; Table 3).

As the LMM analysis demonstrated significant associations between BMI, carbohydrate intake, and postprandial CO_2_ response, these parameters were then divided into 3 categories each. BMI was classified as healthy (18.5-25 kg/m^2^), overweight (25-30 kg/m^2^), and obese (≥30 kg/m^2^), while carbohydrate intake was classified as low (0-30 grams), medium (30-60 grams), and high (≥60 grams).

**Figure 3:**
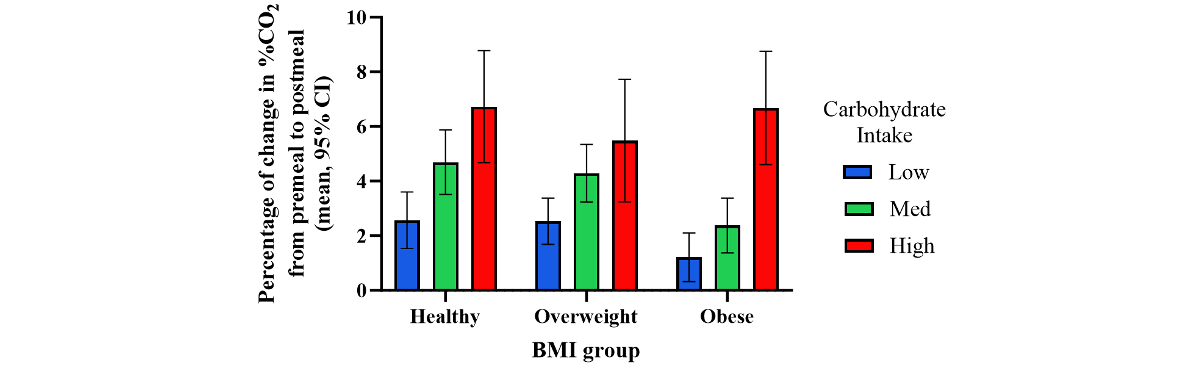
Carbon dioxide (CO_2_) response to different carbohydrates intake in different BMI categories (N=2607). Results represent mean (95% CI).

To examine how the CO_2_ response differed between each BMI category for each carbohydrate intake class, we conducted a 2-way ANOVA. In this analysis, only the last session of each user was used to eliminate the effect within the same user’s sessions. In agreement with the results from the LMM, this analysis revealed a significant effect of users’ BMI category (*F*_2,2569_=5.88; *P*=.003) and level of carbohydrate intake (*F*_2,2569_=24.23; *P*<.001), where users with obesity tended to have a lower postprandial CO_2_ response than those with healthy BMI, and high consumption of carbohydrates resulted in a higher CO_2_ response (Figure 3). Nevertheless, users with high BMI had similar elevated CO_2_ response as overweight and healthy users when consuming meals high in carbohydrates.

**Table 3:** Determinants of CO_2_ response to food intake (N=6671).

Variables	β (95% CI)	*z* statistics	*P* value
Intercept	5.221 (3.242 to 7.199)	5.172	<.001
BMI	–0.112 (–0.156 to –0.069)	–5.026	<.001
Age	–0.021 (–0.049 to 0.007)	–1.483	.14
Gender	–0.612 (–1.324 to 0.100)	–1.683	.09
Carbohydrates	0.046 (0.034 to 0.058)	7.496	<.001
Fat	0.018 (–0.001 to 0.038)	1.816	.07
Protein	0.016 (–0.002 to 0.035)	1.755	.08

## Discussion

### Principal Findings

This study showed how the Lumen device can detect the %CO_2_ response to meals with a different macronutrient composition (mixed meals). Notably, this response is influenced by the carbohydrate consumption in each meal as well as the BMI of the user. Lumen’s measured exhaled %CO_2_ was higher when carbohydrate intake was higher, in accordance with previous studies describing the association between RER and carbohydrates [11,14,15,18]. In addition, it reveals metabolic inflexibility in individuals with obesity as they show a reduced %CO_2_ response compared with healthy individuals, which again is in accordance with the literature on metabolic flexibility [8,9,26].

### Comparison With Previous Work

A recent prospective study has shown that a high-carbohydrate diet results in an elevation of Lumen’s %CO_2_ compared with a low-carbohydrate diet [18]. In this retrospective analysis of Lumen users, we show similar results, as %CO_2_ is elevated when carbohydrate consumption is increased, which highlights Lumen’s ability to detect changes in carbohydrate intake. Although previous studies that included high-fat and high-protein consumption resulted in elevated RER [14,27,28], we did not observe this in this study. Further study with a larger sample size is warranted to check for the effect of dietary fats and proteins on exhaled %CO_2_.

The duration of %CO_2_ elevation has shown a consistent plateau, spanning from 45 minutes postmeal to the last checkup at 210 minutes, where a subsequent decline was observed. Lumen users often analyze their postmeal breath between 60 and 120 minutes, so understanding how %CO_2_ responds over longer periods is challenging. Woerle et al [29] showed carbohydrate oxidation up to 360 minutes after meal consumption, long after glucose and insulin levels in the plasma peaked. Future prospective studies should address when %CO_2_ decreases after a mixed macronutrient meal and how it changes compared to other metabolites.

The impact of postprandial measurement of glucose levels on metabolic disease prevention has been discussed in previous studies [30]. Novel technological advancements have made it available to consumers, mostly with recent developments in continuous glucose monitoring (CGM) [31]. Other metabolic indicators, such as insulin, lipids, metabolomics, and indirect calorimetry, have also been used to assess metabolic flexibility and were found to be predictive of body weight, metabolic syndrome, T2D, and CVDs [11,25,32,33]. As of today, CGM devices need replacing every 2 weeks and are somewhat invasive, whereas most of the other aforementioned measurements can only be performed in a laboratory setting. In contrast, the Lumen device can be used at home and measures an individual’s metabolic state with a simple breath maneuver, which was found to be in agreement with the RER measured in the metabolic cart [17]. Moreover, compared to the metabolic cart, the portability of the Lumen device enables the collection of CO_2_ measurements from a large number of participants after consuming mixed macronutrient meals, which provides a higher level of external validity.

In addition to the effect of carbohydrate consumption, BMI was also found to be a key parameter in the postprandial %CO_2_ response, as a higher BMI resulted in a reduced response. These results are in accordance with the literature from the metabolic cart, where participants with obesity and T2D showed low ΔRER after insulin stimulation [9,10].

In this analysis, the majority of participants were women (2137/2607, 81.97%), in line with similar nutrition and weight management studies finding that women participate 3 times more often than men [34,35]. Age and gender were both statistically insignificant in their effect on the postprandial %CO_2_ response in the LMM, while some studies mention that metabolic flexibility is reduced with age [36]. Although progesterone is directly associated with CO_2_ [37,38], our analysis did not reveal any influence of gender on metabolic flexibility, possibly since women may have been in different phases of their menstrual cycle or at menopause. Future studies should investigate the effects of gender and the menstrual cycle on Lumen’s %CO_2_.

This study shows the potential of the Lumen device and mobile app to improve outcomes for people with metabolic disorders. There is increasing evidence showing that mobile health technologies can improve metabolic outcomes, in particular for lifestyle modifications in T2D and weight loss [39-41]. While most of these technologies incorporate a mobile app only, many studies have shown the benefits of an app accompanied by a device capable of providing real-time feedback on how those lifestyle modifications affect their measurements, which can improve engagement with them [42-45]. In a recent pilot clinical study, Lumen device and app usage improved several metabolic parameters in prediabetic patients after 3 months [46]. In light of these findings, the potential public health implications of mobile and ubiquitous health tools such as Lumen are noteworthy, showing great promise for addressing the epidemic of metabolic dysfunction across the globe in the near future.

### Limitations

Despite the large data set of real-world evidence that was used in this analysis, several limitations need to be mentioned. First, due to its retrospective and observational nature, we cannot identify causal relationships between any of the variables in this study. Second, since this analysis is based on real-world data, the macronutrient consumption of each meal may be miscalculated or incomplete (recall bias), and a misuse of the device might occur as well. Moreover, the heterogeneous composition of the meals makes it difficult to determine if other factors in the meal might influence the %CO_2_ as well, in particular the type of carbohydrate and the fiber composition. In addition, Lumen users’ macronutrient intake might not be representative of most typical diets, as the Lumen app guides them toward a specific diet, primarily low in carbohydrate, and with most of them on a weight loss journey. As this study has specific user characteristics, caution should be exercised in interpreting these conclusions for a broader audience. Furthermore, this analysis did not consider the fasted %CO_2_ levels, which, together with the postprandial levels, could also be useful in the metabolic flexibility assessment [47]. Nonetheless, we believe that due to the nature of the outcome variable, which is the percentage of change from premeal to postmeal, it might have limited to no effect on the current results.

Lastly, postprandial %CO_2_ might be affected by other confounding variables, including preexisting conditions; medication use; dietary restrictions; the menstrual cycle; and lifestyle factors such as stress, sleep, and physical activity, which were not controlled for in this study. Future studies, both prospective and retrospective, should aim to address these limitations, particularly through controlled prospective investigations involving diverse populations and retrospective analyses that comprehensively consider potential covariates.

### Conclusions

In conclusion, Lumen’s ability to evaluate metabolic flexibility following mixed meals was demonstrated through this retrospective analysis of postprandial exhaled %CO_2_, in which increased %CO_2_ was specific to carbohydrate consumption but not to fat consumption. Furthermore, a moderate %CO_2_ response was also observed among users with a high BMI, which suggests metabolic inflexibility among this group. As such, we propose Lumen as an accessible tool allowing individuals to make informed dietary choices conducive to metabolic health.

## Abbreviations

BRANY: Biomedical Research Alliance of New York
CGM: continuous glucose monitoring
CO_2_: carbon dioxide
CVD: cardiovascular disease
IRB: institutional board review
LMM: linear mixed model
RER: respiratory exchange ratio
SBER: Social, Behavioral, and Educational Research
T2D: type 2 diabetes
USDA: United States Department of Agriculture
